# Ligustrazine Inhibits Cartilage Endplate Hypertrophy via Suppression of TGF-*β*1

**DOI:** 10.1155/2016/1042489

**Published:** 2016-08-02

**Authors:** Shufen Liu, Bizeng Zhao, Huipeng Shi, Qianqian Liang, Yishan Fu, Zhu Yang, Leqin Xu, Yongjun Wang, Qin Bian

**Affiliations:** ^1^Department of Orthopaedics & Traumatology, Longhua Hospital, Shanghai University of Traditional Chinese Medicine, No. 725 South Wanping Road, Shanghai 200032, China; ^2^Institute of Spine, Shanghai University of Traditional Chinese Medicine, No. 725 South Wanping Road, Shanghai 200032, China; ^3^Department of Orthopaedics, Sixth People's Hospital, Jiaotong University, No. 600 Yi Shan Road, Shanghai 200233, China

## Abstract

CEP hypertrophy is one of the characteristics of intervertebral disc degeneration (IDD). LIG exerts a protective effect on IDD in animal model. The effect of LIG on CEP hypertrophy is further investigated in the present study. Cells were isolated from hypertrophic samples obtained from patients during vertebral fusion surgery. Cellular proliferation and the expression of type I collagen (Col I) and TGF-*β*1 were tested. In the bipedal rats, the edges of the CEP and the sizes of noncartilaginous outgrowth, as well as the expression of osteogenic markers, Col1a, ALP, Runx2, and TGF-*β*1, were detected. Within two passages, the condensed hypertrophic CEP cells exhibited osteogenic capacity by bony-like nodules and ALP positive staining, along with increased expression of Col I and TGF-*β*1. LIG inhibited proliferation of CEP cells and downregulated the expression of Col I and TGF-*β*1* in vitro*. Furthermore, LIG attenuated CEP hypertrophy on the lumbar spine of bipedal rats by reducing Col1a, ALP, Runx2, and TGF-*β*1 mRNA expression and TGF-*β*1 distribution* in vivo*. We concluded LIG exerted a preventive effect on CEP hypertrophy via suppression of TGF-*β*1 levels. This information could be used to develop alternative therapeutic methods to treat spinal CEP hypertrophy.

## 1. Introduction

Cartilaginous endplate (CEP) hypertrophy, a distinct feature of intervertebral disc degeneration (IDD), is considered as the early stage of osteophyte formation, which is adaptive bone remodeling in response to progressive changes in the mechanical environment [1, 2]. More evidence has shown the degree of IDD to be positively correlated with the size of bony outgrowths or calcified hypertrophy [3, 4].

Although therapies suitable to the relief of pain and improvement of function in IDD patients, such as nonsteroidal anti-inflammatory drugs (NSAIDs), nonopioid analgesics, opioid analgesics, glucosamine, and chondroitin, have been studied, only a few have been verified to have an effect on CEP hypertrophy or following osteophyte development. For example, long-term estrogen replacement therapy (ERT) was found to reduce the prevalence of abaxial osteophytes in the lateral tibial plateau but not the medial plateau in an ovariectomized (OVX) cynomolgus macaques model of naturally occurring OA [5]. Bisphosphonates may have both chondroprotective and osteophyte-preventive effects, as determined by analysis of data from a randomized controlled trial [6]. The level of beta-carotene was low in elderly patients with lumbar osteophytes, suggesting that this antioxidant had an inhibitory on lumbar spine degeneration [7]. However, newer and safer drugs that are also effective in the prevention of CEP hypertrophy and osteophytes development are still pursued.

Ligustrazine (LIG), extracted from* Ligusticum chuangxiong* hort, has been reported to have a therapeutic effect on OA, and one study observed few adverse effects during 5 weeks for treatment and 3 months of follow-up [8]. In our previous studies, we found LIG exerts a preventive effect on IDD in animal model [9]. Moreover, the qishejingkang recipe (QSJKR), of which LIG is the major constituent, was found to decrease the activity of alkaline phosphatase (ALP) in degenerated vertebral discs and inhibit CEP hypertrophy [10]. Therefore, we entered the present project expecting LIG to have an inhibitory effect on CEP hypertrophy.

To determine the inhibitory effect of LIG* in vitro*, we collected hypertrophic CEP samples from patient during vertebral fusion surgery. Cells were isolated and cultured. Cellular proliferation and the mRNA and protein levels of Col1a and TGF-*β*1 were assayed using MTT, qPCR, immunofluorescence, and western blot analysis. To investigated whether LIG could attenuate CEP hypertrophy* in vivo*, we employed a CEP hypertrophy rat model as previously reported [4, 11]. Safranin O fast green staining and morphometry were performed to determine the edges of the CEP and the sizes of noncartilaginous outgrowths. The mRNA expressions of type I collagen (Col1a), ALP, runt-related transcription factor 2 (Runx2), and TGF-*β*1 were detected by qPCR. TGF-*β*1 protein distributions were observed using immunohistochemistry. Our results revealed that LIG has a preventive effect on CEP hypertrophy via the suppression of TGF-*β*1 levels. This information could be used to develop alternative therapeutic for spinal degeneration disease accompanied by CEP hypertrophy.

## 2. Materials and Methods

### 2.1. Cell Culture and Drug Preparation

Samples were obtained from ten patients during vertebral fusion surgery (with previous oral informed consent, approved by Ethics Committee of the Sixth People's hospital, Shanghai, CN). The samples were washed with Dulbecco modified Eagle's medium with high glucose content (DMEM-HG, Biowest, Nuaillé, France). Then the tissues were dissected into 1-2-mm^3^ pieces and cultured in a medium containing DMEM-HG, 20% fetal bovine serum (FBS, Gibco, Langley, OK, USA), and 1% penicillin-streptomycin (Gibco, Langley, OK, USA).

Ligustrazine phosphate (purity > 99%, MW: 252.21) was purchased from the Chinese Medicines and Biological Products Institute (Beijing, CN). The solutions of ligustrazine phosphate were prepared in dimethylsulfoxide (DMSO, Sigma, USA) for the* in vitro* experiments. The final concentration of DMSO was 0.1%.

### 2.2. Animal Models and Drug Administration

Male Sprague-Dawley (SD) rats aged 1 month (*n* = 30), were provided by the Shanghai Laboratory Animal Center (SYXK2003-0002, Science and Technology Commission of Shanghai Municipality gave approval for this experimental study on animals), were randomly divided into Sham, Vehicle (Veh), and LIG groups. In the Veh and LIG groups, rats were forced to stand up by forelimbs surgery as previously described [12]. In the Sham group, the rats did not receive any treatment and were maintained in standard cages. Eight months after the surgery, ligustrazine hydrochloride (Nanning Maple Leaf Pharmaceutical Co., Ltd, CN (lot number: 051125)) was intraperitoneally injected into rats of LIG group (16 mL/kg·d, 10 mL sterile saline: 40 mg ligustrazine hydrochloride) while sterile saline of equal volume was injected into rats of Veh group once a day for one month. Rats (*n* = 10) in each group were euthanized at 9 months after the surgery. Their lumbar spines were dissected for analysis.

### 2.3. ALP Assay

The cells were fixed with 10% formalin and stained with 1-Step*™*NBT-BCIP (Pierce, USA) for 30 minutes. Lyons blue represents the positive staining. The images were scanned (CanoScan 8800F, Japan).

### 2.4. MTT Detection

Passage III hypertrophic CEP cells were cultured in 96-well plates at 1 × 10^5^/mL with 200 *μ*L medium (the medium contained LIG and 20% FBS) for 8 wells per group. After being cultured for 1, 5, 9, and 13 days, 20 *μ*L of 5 mg/mL MTT reagent was added to each well and they were incubated for 4 hours at 37°C. Media were removed, followed by adding 150 *μ*L MTT solvent.

The OD values were tested at 590 nm after shaking for 15 min. A cellular growth curve was produced to reflect the OD values.

### 2.5. qPCR

RNA was extracted from L_1-2_, L_2-3_, and L_3-4_ marginal discs (noncartilaginous outgrowth) or hypertrophic CEP cells treated by LIG for 48 hours using 1 mL TRIzol reagent (Sigma, St. Louis, MO, USA) according to the manufacturer's instruction. Cells/tissues were directly processed following RNA preparation employing the PURE Prep Kit protocol. One microgram of total RNA was reverse-transcribed using the Advantage RT-for-PCR Kit (Qiagen, Valencia, CA, USA) according to the manufacturer's instructions. Freshly transcribed cDNA (1 *μ*L) was employed for qPCR using SYBR Green (Bio-Bad, Hercules, CA, USA) to monitor DNA synthesis with specific primers (Tables 1 and 2) designed by TaKaRa Biotechnology Co. Ltd. Gene expression was normalized to the housekeeping gene *β*-actin. PCR products were subjected to melting-curve analysis, and data were analyzed and quantified with the RotorGene 6.0 analysis software.

### 2.6. Western Blotting

Cells were cultured in basal medium or 10^−7^ M LIG for 10 days. At the end of the study, the cells were washed with PBS, scraped, and resuspended in lysate (Beyotime, Hangzhou, CN) for 1 hour on ice. The cell lysates were centrifuged at 12,000 rpm for 10 minutes at 4°C. The supernatant was transferred to a new microcentrifuge tube, and protein concentration was measured by Bradford protein assay (Beyotime, Hangzhou, CN). Proteins were added with 4x sample buffer (0.01% bromophenol blue, 0.125 M Tris, pH 6.8, 10% glycerol, and 2% SDS) and denaturized at 95°C for 10 minutes. Equal amounts of protein (50 *μ*g/lane) were solubilized in Laemmli sample buffer and loaded onto a mini-SDS-PAGE system. Following electrophoresis, the proteins were transferred to a polyvinylidene difluoride (PVDF) membrane (Millipore, Temecula, CA, USA) using a Bio-Rad wet transfer system. Protein transfer efficiency was verified using prestained protein markers. The membranes were then blocked with 5% nonfat milk for 1 hour at room temperature and subsequently incubated overnight at 4°C with TGF-*β*1 antibodies directed against the protein (1 : 500, Lifespan BioSciences, Seattle, WA, USA). Chemiluminescence images were obtained by application of LAS-4000 (Fujinfilm, Tokyo, Japan) and Image Reader LAS-3000 software in precision mode.

### 2.7. Safranin O Fast Green Staining

The lumbar spines of the rats were fixed in 4% paraformaldehyde for 24 hours and washed for 2 hours. Then they were decalcified in 20% EDTA for 4 weeks, and the fluid was changed once a week. Lumbar spines were dehydrated and embedded in paraffin wax. At least four consecutive sections of 7 *μ*m in thickness were obtained from the sagittal planes and stained with safranin O and fast green. A morphometric study was performed using an image auto-analysis system (Olympus BX50; Japan). L_4_ were examined.

### 2.8. Immunohistochemical Analysis and Cytoimmunofluorescence

Sections were pretreated and incubated with antibody against TGF-*β*1 (1 : 100, Cell Signaling Inc. MA, USA) overnight at 4°C and incubated with biotinylated goat anti-rabbit-IgG for 15 minutes at 37°C. This was followed by streptavidin-HRP for 10 minutes at 37°C. Bound complexes were visualized using 3,3′-diaminobenzidine (DAB) reagent, counterstained with hematoxylin, dehydrated, and mounted with gummy for immunohistochemical assays. The data were quantified using a medical image management system (Cmias, CN).

In cytoimmunofluorescence assays, samples were incubated with antibody against Col I (1 : 500, Abcam Ltd. Cambridge, UK) overnight at 4°C. They were then incubated with the secondary fluorescent antibodies for 1 hour at 37°C and counterstained with 2-(4-amidinophenyl)-6-indolecarbamidine dihydrochloride (DAPI). Light (Olympus DP71, Japan) and fluorescence microscopes (Leica DM3000B, Germany) were used.

### 2.9. Statistical Analyses

The data are expressed as means ± SE, and statistical significance was calculated using one-way ANOVA followed by a post-hoc LSD test (homogeneity of variance) and Tukey's test (heterogeneity of variance) using SPSS software (SPSS Inc, Chicago, USA). The significance level was defined as *p* < 0.05.

## 3. Results

### 3.1. Isolation and Characteristics of Cells from Hypertrophic CEP Samples

We collected hypertrophic CEP samples from patients who underwent vertebral fusion surgery and cultured these tissues of small pieces. Hypertrophic CEP-derived cells could be seen to slough off from the side of the tissue on day 16.

**Figure 1:**
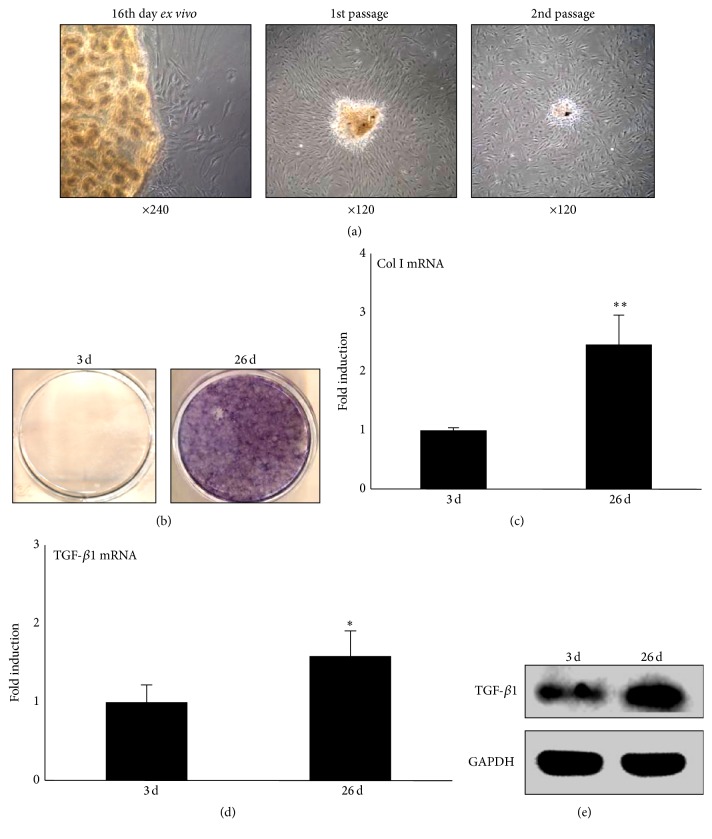
Characteristics of hypertrophic CEP cells from patient samples. (a) Hypertrophic CEP-derived cells could be seen sloughing off the tissue on day 16. After the first and second passages, several bony-like nodules could be seen although the CEP pieces had been removed off. (b) Cells with low concentration were negative for ALP-staining on day 3. On day 26, the condensed cells spontaneously showed strong positive ALP staining. (c, d) mRNA levels of (c) Col1a1 and (d) TGF-*β* were detected by qPCR. (e) TGF-*β* protein level was tested by western blot. Each column represents the mean ± SE of three independent experiments. ^*∗*^ *p* < 0.05, ^*∗∗*^ *p* < 0.01.

To obtain adequate cells for experiments, we identified that passage had little effect on cellular morphology. We found that these cells showed polygonal and spear-like morphology following initial passage. After the first and second passages, several bony nodules could be seen, even though the tissue pieces had been removed off. Cells retained these shapes and began to grow from or up to the nodules (Figure 1(a)).

As these bony nodules were seen when the cells reached to a high concentration, we hypothesized that hypertrophic CEP cells have osteogenic capacity when they undergo condensation as hypertrophic chondrocytes do during primary ossification [13]. To test this hypothesis, ALP assay was used. The results showed negative ALP-staining for cells of low concentration on day 3 of culture. However, the condensed cells spontaneously showed strong, positive staining on day 26 of culture, indicating these cells had an osteogenic potential (Figure 1(b)).

To further identify the characteristics of hypertrophic CEP cells in these two states: low concentration and high concentration (condensation), we detected function protein of osteoblasts: type I collagen. The mRNA level of Col1a1 of the condensed cells (26 d) was higher than that in the cells at low concentration (3 d) (*p* < 0.01) (Figure 1(c)).

Then, we tested TGF-*β*1 expression of hypertrophic CEP cells since TGF-*β*1 has been reported to contribute to CEP hypertrophy [14]. We found condensed cells expressed more TGF-*β*1 and protein than cells of low concentration did (Figures 1(d) and 1(e)).

Taken together, the results suggested that condensed hypertrophic CEP cells have osteogenic capacities.

### 3.2. Effects of LIG on Proliferation and Col1a and TGF-*β*1 Expression in Hypertrophic CEP Cells

**Figure 2:**
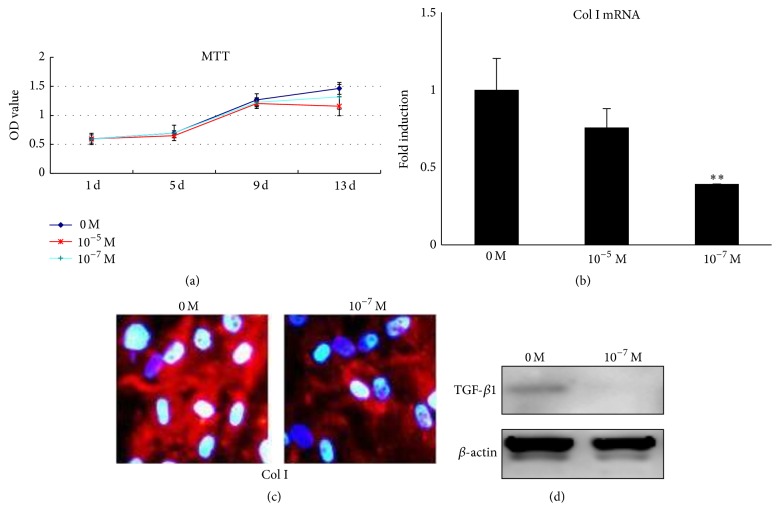
Effects of LIG on cell proliferation and Col1a and TGF-*β* expression* in vitro*. (a) MTT assays showed that passage-III hypertrophic CEP cell proliferation decreased on day 13 after LIG treatment at two doses compared to the control (0 M). The inhibitory effect of LIG was more pronounced at a dose of 10^−5^ M than at 10^−7^ M. (b–d) Condensed cells were treated with LIG. (b) Col1a1 expression was significantly decreased by LIG of 10^−7^ M. LIG also showed inhibitory tendency at a dose of 10^−5^ M with no statistical significance. (c) Cytoimmunofluorescence results showed attenuated positive staining for Col I in the extracellular matrix with LIG of 10^−7^ M compared to the nontreatment. (d) Western blot analysis of TGF-*β* expression by 10 days of LIG treatment at a dose of 10^−7^ M. Each column represents the mean ± SE of three independent experiments. ^*∗∗*^ *p* < 0.01* versus* 0 M.

To investigate if LIG has effect on proliferation of hypertrophic CEP cells, we did MTT assays. The results showed a decrease in cell proliferation by LIG treatment for 13 days at two doses. The inhibitory effect of LIG was more pronounced at a dose of 10^−5^ M than 10^−7^ M (Figure 2(a)).

Then, we tested the effect of LIG on Col1a and TGF-*β*1 expression of condensed hypertrophic CEP cells. As a result, LIG treatment decreased Col1a1 expression of the condensed cells at the dose of 10^-7 ^M (*p* < 0.01). A dose of 10^−5^ M LIG also showed a decreased tendency but no significant differences were found (Figure 2(b)). The results have been identified by cytoimmunofluorescence examination, which demonstrated attenuated positive staining for Col I in the extracellular matrix of hypertrophic CEP cells by treatment with 10^−7^ M LIG (Figure 2(c)). In addition, the protein level of TGF-*β*1 was significantly suppressed with LIG at same dose by western blot analysis (Figure 2(d)).

### 3.3. The Effects of LIG on CEP Hypertrophy on Lumbar Spine of Rats

**Figure 3:**
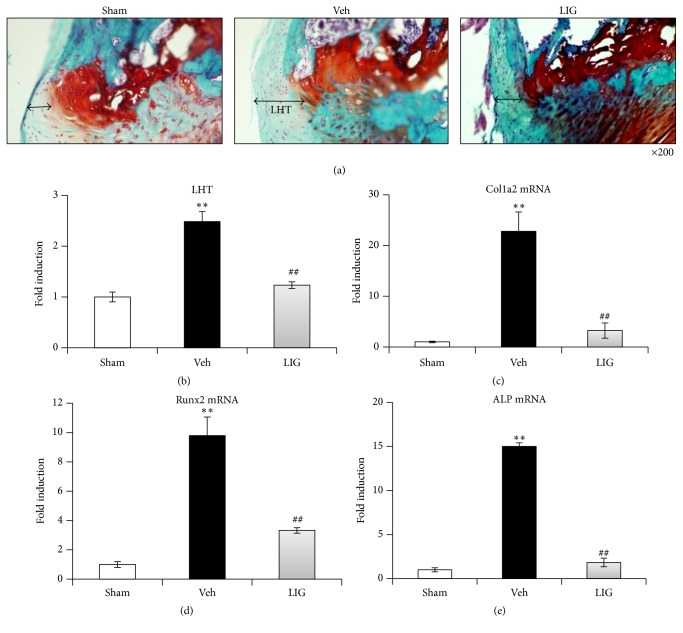
Effects of LIG on CEP hypertrophy of the lumbar spine in bipedal rats. (a, b) Representative images of Safranin O fast green staining of L_4_ section showed a significant increase in OFT at the marginal disc of bipedal rats. LIG partially reduced the noncartilaginous outgrowth. (c–e) The expression of the three osteogenesis-related markers: (c) Col1a2, (d) ALP, (e) Runx2 in the Sham, Veh, and LIG groups. Each column represents the mean ± SE, ^*∗∗*^ *p* < 0.01* versus* Sham, and ^##^ *p* < 0.01* versus* Veh.

To investigate the effect of LIG on CEP hypertrophy* in vivo*, we employed a rat model as previously reported to induce CEP hypertrophy [14]. After 1-month treatment of LIG, the lumbar spines of rats were collected for examination. The distance between marginal articular cartilage (red staining) and marginal noncartilaginous outgrowth (green staining) was measured, which is defined as the thickness of limbic hypertrophy (LHT) as described previously. Results showed a significant increase in LHT in rats kept in an upright posture for 9 months. LIG was found to significantly decrease LHT (Figures 3(a) and 3(b)).

To further examine the inhibitory effects of LIG on CEP hypertrophy through inhibition of osteogenic function, three osteogenesis-related markers were detected. Our qPCR studies demonstrated that the expression levels of the three osteogenesis-related markers, Col1, ALP, and Runx2, increased in Veh samples relative to Sham. However, LIG treatment caused significant decreases in expression of these three genes as compared to Veh treatment (Figures 1(c)–1(e)).

### 3.4. The Effects of LIG on the TGF-*β*1 Expression in CEP Tissue of Rats

**Figure 4:**
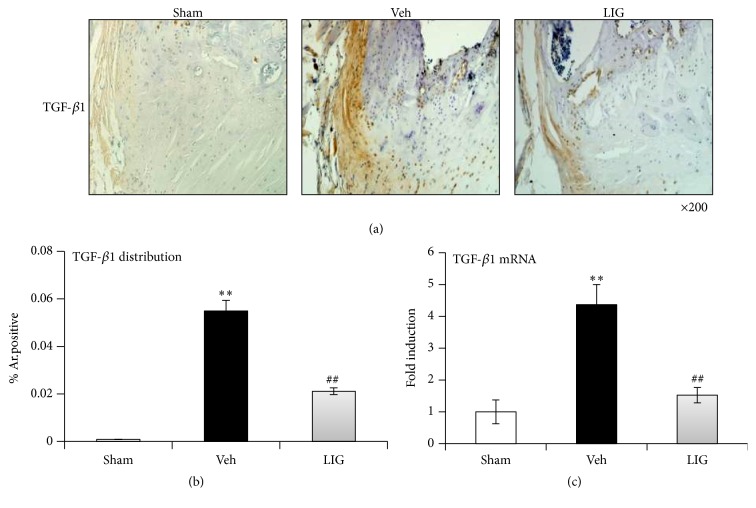
LIG suppresses TGF-*β*1 distribution and expression in CEP tissue of bipedal rats. (a) Immunohistochemical staining demonstrated stronger positive matrix staining for TGF-*β*1 (brown) in the marginal area in the Veh group compared to that in the Sham group. LIG treatment weakens the positive staining for TGF-*β*1. (b) Quantitative data of (a). (c) LIG reduced the increased mRNA level of TGF-*β*1 in bipedal rats by qPCR. Each column represents the mean ± SE, ^*∗∗*^ *p* < 0.01* versus* Sham, and ^##^ *p* < 0.01* versus* Veh.

As increased TGF-*β*1 expression was found* in vitro*, we further confirm it* in vivo*. Strong positive matrix staining for TGF-*β*1 was detected in the marginal area in the Veh group. This was much weaker in the Sham and LIG groups (Figures 4(a) and 4(b)). TGF-*β*1 mRNA levels showed the same tendency as TGF-*β*1 protein levels in all three groups (*p* < 0.01) (Figure 4(c)).

## 4. Discussion

In this study, we revealed the inhibitory effect of LIG on CEP hypertrophy via suppression of TGF-*β*1 expression both* in vivo* and* in vitro*. Our study is the first to report a preventive effect of LIG. From these results, it seems suitable as an alternative therapeutic method for spinal degenerative disease accompanied by CEP hypertrophy.

Pathologic characteristic manifestations of spinal degeneration and CEP hypertrophy and following osteophyte formation are commonly seen. An experimental model of cervical spondylosis in rabbits showed a process of osteophyte formation involving endochondral calcification and ossification established by resection of the cervical supraspinous and interspinous ligaments and detachment of the posterior paravertebral muscles from cervical vertebrae [10, 15]. Puncture of a lumbar disc based on the presence of nucleus pulposus contributed to the formation of disc nodules and osteophytes [16]. It was reported by Gloobe that osteophyte formation occurred in experimental bipedal rats [11]. In our previous study, calcified hypertrophy, considered to be osteophyte formations low rate of progression, was induced in the regions of lumbar vertebral column by prolonged upright posture [4, 17]. This model was chosen for the* in vivo* observation in this project. Because the gradually increased thickness of the marginal CEP could be observed over time (at 5, 7, and 9 months after the surgery), decreases were taken to indicate preventive and therapeutic effects of LIG in the parameters of LHT and changes in the expressions of three osteogenesis-related markers at mRNA levels: Col1, ALP, and Runx2 at 9 months after the surgery with one month of treatment.

Transforming growth factor-beta 1 (TGF-*β*1) has been found to play a crucial role in endplate hypertrophy and bony outgrowths [18]. The expression of TGF-*β*1 mRNA is higher in the early-mid stages of osteophyte development [19]. Overexpression of TGF-*β*1 can induce local outgrowths similar to those observed under experimental conditions [20–23]. Inhibition of endogenous TGF-*β* can nearly completely prevent hypertrophy development by scavenging soluble TGF-*β*-RII [24, 25]. However, synovial lining cells, such as macrophages, might contribute to TGF-*β*1-mediated bony outgrowths [23, 26, 27]. Consistent with several reports stating that TGF-*β*1 contributes to the process of osteophyte formation, our findings have shown TGF-*β*1 to be distributed in hypertrophyic CEP tissues and cells. To show this, we used the same immunochemical methods that Uchino et al. used to locate TGF-*β*1 in the superficial cells of hypertrophic cartilage [28, 29].

This study has some limitations. First, several kinds of cells have been observed in newly formed hypertrophic CEP tissue. These include fibroblastic mesenchymal cells, fibrochondrocytes, chondrocytes, and osteoblasts [30]. Osteophyte-derived mesenchymal stem cells (oMSCs) have been characterized with regard to their distinct properties of proliferation, differentiation, and immunomodulation [31]. Our results indicated hypertrophic CEP cells have osteogenic potential, which acted as oMSCs. However, we did not isolate different cell types from the hypertrophic CEP tissues. The effects of LIG on specific cell types could not be verified. Second, the data showing that LIG inhibits CEP hypertrophy were obtained from animal models* in vivo* and human hypertrophic CEP samples* in vitro*. Future studies will have to gather clinical evidence.

## 5. Conclusions

In conclusion, LIG exerted a preventive effect on CEP hypertrophy via suppression of TGF-*β*1 levels. LIG can be suggested as an alternative therapeutic method to treat spinal CEP hypertrophy.

## Figures and Tables

**Table 1:** Sequences of primers for rats used in the qPCR.

Genes	Forward primer	Reverse primer	Product length (bp)
*β*-actin	GGAGATTACTGCCCTGGCTCCTA	GACTCATCGTACTCCTGCTTGCTG	150
Col 1*α*2	TCCTGGCAATCGTGGTTCAA	ACCAGCTGGGCCAACATTTC	133
Runx2	CCATAACGGTCTTCACAAATCCT	TCTGTCTGTGCCTTCTTGGTTC	99
TGF-*β*1	TGCGCCTGCAGAGATTCAAG	AGGTAACGCCAGGAATTGTTGCTA	82
ALP	TTGAATCGGAACAACCTGACTGAC	GATGGCCTCATCCATCTCCAC	183

**Table 2:** Sequences of primers for humans used in the qRT-PCR.

Genes	Forward primer	Reverse primer	Product length (bp)
*β*-actin	CCTGTACGCCAACACAGTGC	ATACTCCTGCTTGCTGATCC	211
Col 1*α*1	AGAGGGCAGCCGCAAGAAC	CTGGCCGCCATACTCGAACT	280
TGF-*β*1	CCGACTACTACGCCAAGGA	CTGAGGTATCGCCAGGAAT	247
